# AI-LyD: An AI-Driven System Approach to Combatting Spotted Lanternfly Proliferation Through Behavioral Analysis

**DOI:** 10.3390/insects17030272

**Published:** 2026-03-03

**Authors:** Kevin Zhang

**Affiliations:** Independent Researcher, Annandale, NJ 08801, USA; noble.kevinzhang@gmail.com; Tel.: +1-908-327-5889

**Keywords:** spotted lanternfly (SLF), Lycorma delicatula, pest control, integrated pest management (IPM), machine learning, behavioral research, MAXENT, You-Only-Look-Once (YOLO)

## Abstract

The spotted lanternfly (SLF, *Lycorma delicatula*) is an invasive planthopper causing severe agricultural and environmental damage in 20 U.S. states. SLF control remains constrained by (1) overreliance on broad-spectrum pesticides that harm nearby ecosystems, (2) inefficiency and ecological risk of alternative methods, and (3) underutilization of SLF behavioral traits and artificial intelligence (AI) in IPM. This study introduces AI-LyD, an AI-driven IPM framework integrating behavioral ecology, predictive modeling, image-based detection, and low-cost physical controls. Incorporating SLF behavioral constraints, including cold-exposure requirements for egg hatching, into ecological models improved prediction accuracy (AUC = 0.821, Sensitivity = 0.888, Kappa = 0.642) and reconstructed SLF distributions consistent with current proliferation trends. A YOLO-based detection model leveraging SLF clustering behavior improved identification accuracy from 84% to 96% and reduced false positives from 42% to 8% in real-world drone-collected imagery. Exploiting SLF crawling, jumping, and hydrophobic behaviors, the novel Aquabex water-moat device with an optimized 60° opening trapped 85% of Stage I–IV nymphs and reduced adult invasions by 67%, at an estimated cost below USD $0.50 per unit. Field deployments across four locations in Hunterdon County, New Jersey, achieved a 91% population reduction (95% CI: 90.1–92.0%). Together, these results establish AI-LyD as the first operational, scalable SLF IPM system, and this paradigm can be applied to controlling other invasive species.

## Simple Summary

The spotted lanternfly (SLF, *Lycorma delicatula*), is an invasive insect causing serious damage to agricultural industries and natural ecosystems. Current control methods either harm beneficial pollinators and pollute the environment, are expensive to maintain, or are inefficient at larger scales. Many applicational technologies (ex. artificial intelligence) used in combatting SLF also fail to utilize SLF’s unique behaviors. This study introduces AI-LyD, a novel integrated pest management (IPM) framework that combines insect behavior, artificial intelligence, and low-cost physical controls to manage SLF populations. The system predicts where SLF are most likely to spread, detects them automatically in drone-collected images, and reduces the bugs’ movement through the novel Aquabex water-barrier trap, which costs less than 50 cents per unit. When deployed across trial locations, AI-LyD reduced SLF populations by 91%. This work demonstrates that integrating SLF behavior into AI-based applications and solutions can provide a scalable, sustainable way to control SLF invasions.

## 1. Introduction

### 1.1. Issue Statement

The spotted lanternfly (SLF), *Lycorma delicatula*, is native to Asia, but has spread to other geographic regions via human activity [1,2]. Since the first sightings of SLF in Berks County, Pennsylvania in 2014 [3], SLF have spread to 20 U.S. states and over 200 counties [4,5,6]. New counties and states are added to the list each year, and SLF are expected to invade all the Midwest and Southeast [7]. Although they exhibit strong preference toward *Ailanthus altissima* (Tree-Of-Heaven, TOH) and *Vitis* spp. (grapevines), SLF are reported to feed on over 103 taxa and threaten important agricultural products [8,9,10]. The state of Pennsylvania alone estimates USD $554 million in annual damage from SLF [11].

**Figure 1:**
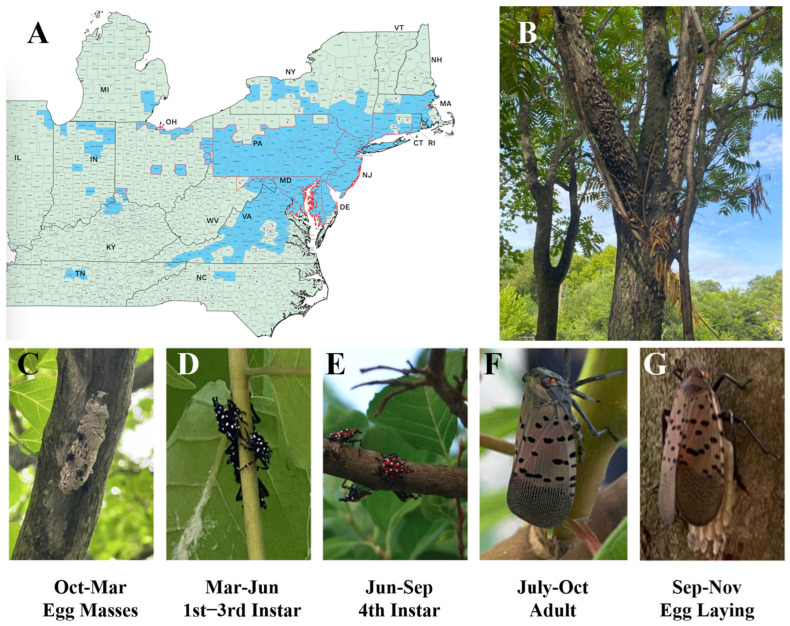
(**A**) SLF distribution map across the eastern U.S. (**B**) SLF infestation on a TOH along the Landsdown Trail, Clinton, NJ, USA. (**C**–**G**) SLF life cycle. Map in (**A**) courtesy of Cornell University College of Agriculture and Life Sciences (CALS).

SLF lay egg masses on tree bark, rocks, or manmade surfaces, hatching in late spring and progressing through four instar stages (I–IV) (Figure 1D,E) before maturing into winged adults (Figure 1F) and laying eggs in the fall (Figure 1G) [12,13]. Due to their mobility, systemic elimination is especially difficult [14,15]. SLF exhibit destructive feeding behavior in large clusters and characteristic hopping-and-gliding movement [14,16]. SLF also excrete honeydew that promotes sooty mold growth and further stresses plants [17].

### 1.2. Current Solutions

Several solutions have been proposed to reduce SLF populations. Pesticides remain the most effective and widely used approach, but introduce harmful chemical contaminants that pose significant risk to human and ecological health, and damage non-target species, including pollinators such as the western honey bee (*Apis mellifera*) [18,19,20,21,22]. SLF-specific biocontrol, such as the fungus *Beauvaria bassiana* and the parasitic wasp *Anastatus orientalis*, have also been explored, though their long-term ecological impact remains uncertain [23,24,25]. SLF management efforts also leverage public participation, including citizen-reporting platforms and public awareness programs [26,27,28]. However, these efforts depend on voluntary action and often fail to cover less accessible environments, and the rapid reproduction of SLF outpaces labor-intensive civilian efforts.

SLF traps also play a central role in population suppression and monitoring [10,29]. Sticky bands exploit SLF’s tendency to crawl upwards and are easy to deploy, but cause non-target mortality in pollinators and birds, require frequent replacement, and are less effective against late-stage nymphs and adults [30,31]. Circle traps, funneling SLF into collection chambers, offer higher capture efficiency with reduced ecological concern, but require precise installation and regular maintenance to prevent SLF from escaping [14]. Recent advancements include design optimization (ex. mesh size, placement) and using live SLF as lures [32].

### 1.3. Literature Review

Given the rapid spread of SLF, accurate modeling is critical for effective management. Mapping tools, public reporting platforms, and ecological prediction models help track and predict SLF distribution [33,34]. While current models account for bioclimatic variables and TOH distribution, current forecasts often contradict real-world SLF distribution patterns [1,7,35,36]. For example, current models predict severe SLF establishment in the U.S. West Coast and Mediterranean regions on account of year-round favorable weather and vulnerable geography, but no sustained SLF presence has been documented, a discrepancy termed the “California Paradox” [7,36]. Many models do not account for SLF behaviors, and do not reflect SLF resilience to climate change-related extremities [37]. Therefore, AI-driven models that incorporate behavioral dynamics and overlooked ecological variables are key to more accurate forecasts of SLF spread.

AI offers promising scalability for invasive species management [10], automating image detection and next-step management. Recent advancements in AI detection incorporate higher-level pattern analysis and behavioral inference to support more intelligent monitoring and decision-making [38]. However, due to the relatively limited and intermittent movement patterns of SLF, existing object detection models for SLF remain at the level of static object detection. For example, the Spotted! model identifies and tracks individual nymphs and adult SLF through spot variations [39]. However, current SLF detection models—trained on SLF photographed in ideal conditions—perform poorly when applied to variable real-world environments. Generalized detection models also similarly suffer from low accuracy when faced with SLF in complex backgrounds and lighting [18,40]. However, collecting images and footage over large areas requires drone surveys or field cameras stationed in natural territory; resulting images and footage thus often have significant noise or limited resolution. To advance IPMs, developing robust models with high identification accuracy even in suboptimal conditions is essential.

Interdisciplinary efforts also offer new approaches to SLF detection and elimination. For example, detector dogs can be trained to identify SLF egg masses [41]. Robotics and AI-based approaches, like The TartanPest project [42] and Artreeficial [43], are innovations supplanting physical traps and combatting SLF. However, these approaches are primarily reactive, addressing only visible SLF populations. Further, they lack a systematic framework for proactive prevention.

Critically, no current approach provides a scalable, cost-efficient, and environmentally sustainable solution to control the rapid spread of SLF. Existing control strategies often underutilize SLF’s distinct biological and behavioral traits, and AI-based tools remain fragmented, with limited integration and reduced accuracy under real-world conditions. Current mitigation strategies lack coordination among approaches. Therefore, the overarching question this research will solve is how SLF’s behavioral traits can be leveraged to construct an adaptive, scalable closed-loop system for prediction, detection, and intervention.

### 1.4. Proposed Solution and Objectives

This research aims to develop AI-LyD, a comprehensive, AI-driven framework integrating behavioral analysis, ecological modeling, and machine learning to systematically address SLF proliferation. Specifically, AI-LyD seeks to (1) expand predictive modeling by incorporating ecological and behavioral determinants of SLF survival, reproduction, and dispersal; (2) enhance detection accuracy through the construction of a large-scale, natural-habitat image database and the utilization of SLF clustering behavior within advanced machine learning architectures; and (3) design Aquabex, a scalable, environmentally sustainable physical control system embedded within an AI-LyD management platform that synthesizes predictive modeling, localized environmental parameters, and real-time detection data to generate comprehensive, IPM-based deployment strategies.

## 2. Materials and Methods

### 2.1. AI-LyD Framework

**Figure 2:**
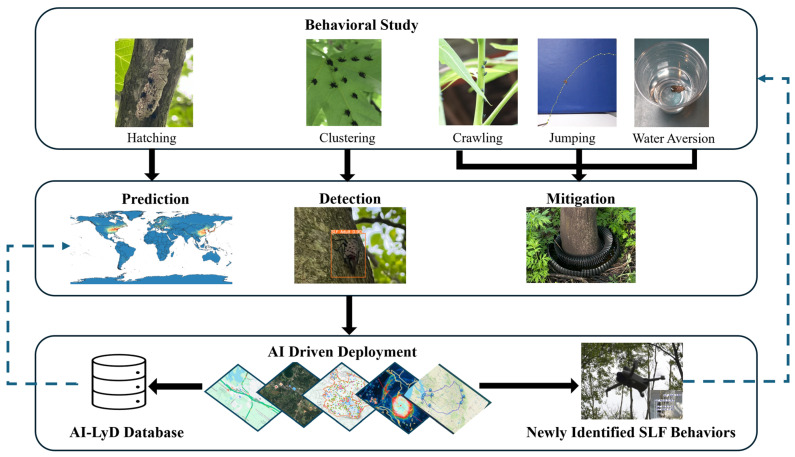
AI-LyD’s system-level architecture for understanding and reducing SLF populations. The framework consists of three phases, each with distinct objectives and research focus.

AI-LyD aims to enhance accuracy in identifying high-risk proliferation regions, improve SLF detection accuracy, and deploy targeted, environment-friendly control measures by leveraging AI-tools and understanding of SLF ethology. AI-LyD’s framework (Figure 2) systematically connects behavioral research and AI implementation, creating a scalable, adaptive solution for managing SLF populations at multiple levels.

### 2.2. SLF Behavior Studies

This phase investigates SLF behavioral traits and leverages these findings to advance subsequent research. Phase 1 consists of the following experiments:

Freezing Period: SLF egg masses were collected from the Tree-of-Heaven (TOH) at Landsdown Trail in Clinton, New Jersey in three batches: on 6 November 2024; on 6 December 2024; and on 6 January 2025. Collected egg masses were tallied (*n* = 153, 135, 141 respectively) and placed in a temperature and humidity-controlled sealed environment (temperature = 25 °C, humidity = 70%). Hatching observations were made daily, with observations concluding after 15 consecutive days of no new hatching activity. Daily temperature data was obtained from the National Centers for Environmental Information for Clinton, New Jersey, the site where SLF eggs were collected.

Clustering and Crawling: Fourth instar SLF nymphs (*n* = 30) were captured, marked with UV-fluorescent dye, and released at the base of the tree. Major branches were labeled, and after 8 h, the location of each SLF was recorded to determine branch preference. UV-marked SLF were filmed to determine their mode of locomotion. Experiments were conducted on *Styrax japonicus* (Japanese Snowbell), *Juglans nigra* (Black Walnut) and *Acer rubrum *(Red Maple) trees in Clinton, New Jersey on 21 July and 22 July 2024. Each experiment was repeated twice on the same tree.

**Figure 3:**
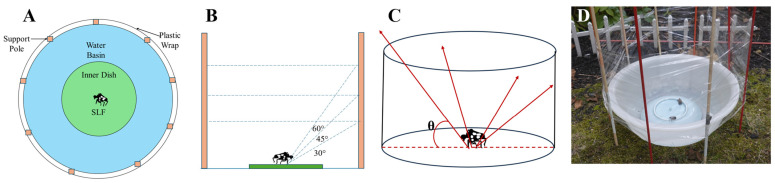
Experimental setup for SLF jumping experiments: (**A**) Bird’s-eye view diagram of testing environment; (**B**) Side view; (**C**) Three-dimensional schematic, with red arrows indicating different possible jumping trajectories; (**D**) Picture of the actual setup.

Jumping: SLF jumping behavior was quantified at all life stages by placing 1st–4th instar nymphs and adult SLF (*n* = 30 per stage) within an experimental enclosure. A circular platform was erected (Figure 3A,B), surrounded by a water moat to prevent escape. A transparent plastic-film barrier encircled the platform at heights correlating to inclinations of 30°, 45°, and 60° relative to the platform surface (Figure 3C,D). SLF were gently prodded with a fine brush to elicit jumping. Each angle condition was tested 30 times, with the take-off trajectory recorded to determine whether the SLF cleared the barrier.

**Figure 4:**
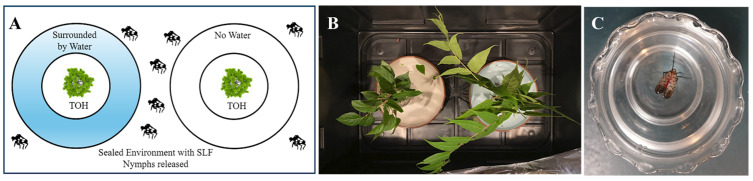
Experimental setup for SLF hydrophobicity tests. (**A**) Experimental Setup for Testing SLF Hydrophobicity. (**B**) TOH branches placed in a sealed environment: one with a water-filled moat and the other with an empty moat. (**C**) SLF survival assay in different solutions: water, 5% ECOS dish detergent, 5% DAWN dish detergent.

Hydrophobicity: SLF nymphs (*n* = 20) from a TOH tree in Clinton, New Jersey were placed in a sealed environment with TOH branches (Figure 4A,B). The experiment was conducted with two options: TOH branches surrounded by a water moat, and one without a moat. The location of the SLF were recorded at the 4, 8, and 24 h mark. In a separate experiment (Figure 4C), SLF were individually dropped into a 5 cm-deep cup of water, and the time to submersion was recorded. The procedure was repeated using 5% eco-friendly ECOS^®^ Dishmate detergent (Venus Laboratories dba Earth Friendly Products, Addison, IL, USA) and 5% conventional DAWN^®^ Free & Clear dish detergent (Proctor and Gamble Company, Cincinnati, OH, USA) solutions to compare time to submersion.

### 2.3. Prediction Model Development

**Figure 5:**
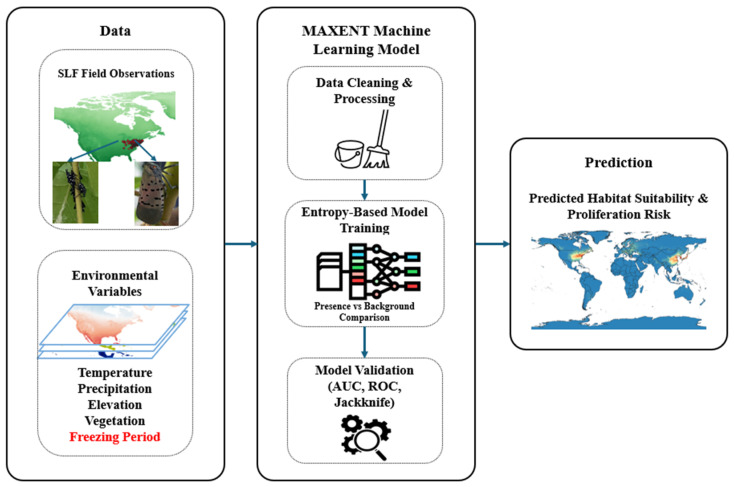
MAXENT data architecture. Environmental layers and occurrence point data are prepared, trained, and validated through statistical analysis (ex. AUC test).

Key survival and proliferation factors influencing SLF survival and proliferation were compiled, and relevant datasets (ex. presence of TOH) and empirical conclusions from behavioral studies (ex. required freezing period for egg hatching, inclement weather) were integrated as environmental data layers (Figure 5). The ML modeling software MAXENT (Maximum Entropy) Version 3.4.4 was chosen for its strong predictive performance and unique ability to model species distribution using presence-only data [44]. All environmental layers were formatted using QGIS with a 10 km resolution. Current SLF distribution was compiled from the iNaturalist and Global Biodiversity Information Facility databases [26,45,46]. A bias file developed from SLF presence points was developed using kernel density estimation to account for spatial sampling biases. The model was set to a maximum of 1000 interactions, with 100,000 background points. A total of 10% of the SLF presence dataset was randomly selected for testing. The model was trained on linear, quadratic, and product features, with the optimal configuration calculated based on performance metrics. Jackknife testing quantified the influence of individual environmental layers on the overall prediction model.

### 2.4. Detection Model Development

In developing an improved SLF detection model, 1250 original SLF images, encapsulating various SLF life stages, were compiled under a variety of lighting conditions and substrate backgrounds to ensure comprehensive dataset coverage for model training. Annotation files were stored in YOLO format (.txt) with each annotation file corresponding to an image of the same file name. Each line within the annotation file includes a class ID (0 for stage 1–3 SLF nymphs, 1 for stage 4 SLF nymphs, and 2 for adult SLF) followed by normalized coordinates for the bounding box. Training, validation, and testing datasets were stratified by visual life stage to preserve proportional class representation. To enhance generalization under field conditions, data augmentation techniques were applied, including brightness and contrast adjustment, hue-saturation variation, JPEG compression, fog simulation, motion blur, random rotation, horizontal and vertical flipping, and resizing.

**Figure 6:**
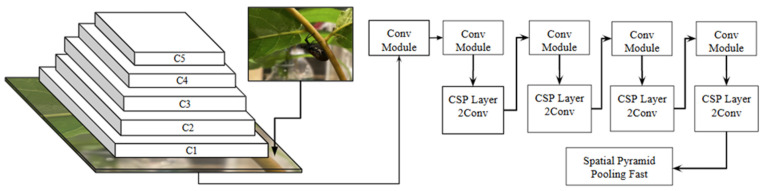
YOLO-v11 data architecture of the backbone. The convolutional layers are responsible for extracting features from the input image, which will be fed into the neck and head of the YOLO model.

For field deployment applications, the YOLO11 (You Only Look Once) architecture was selected for its real-time detection capabilities, high accuracy in a variety of backgrounds, and resource efficiency. The latest version (v11) demonstrates improved accuracy and speed via improved training methods [47]. A YOLO11 nano (YOLO11n) model was trained using image resolution of 640 × 640 over 50 epochs with a batch size of 32. Training was conducted using a standard learning rate of 0.001 for smaller objects. The trained model was then evaluated on two sets of 50 SLF images: one with clusters of SLF, and a “counterpart” image with all but the most noticeable SLF removed from the image Figure 6). The confidence interval was set to 0.10 to capture low-visibility detections in real-world field conditions. Detection rates and false positive percentages were recorded for each set.

### 2.5. Aquabex Fabrication and Installation

**Figure 7:**
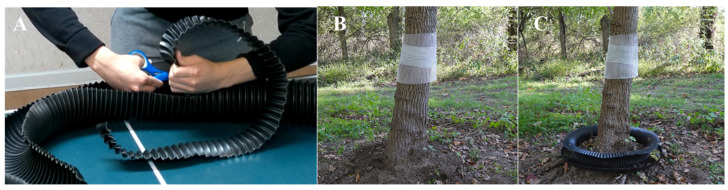
(**A**) Fabrication of Aquabex from a 4-inch drain pipe (**B**) A black walnut tree (Landstown Trail, Clinton, NJ, USA) with sticky band and protection screen without Aquabex; (**C**) the same black walnut tree with Aquabex.

This module of research develops the low-cost “Aquabex” water moat, leveraging SLF hydrophobicity, jumping abilities, and crawling tendencies. The corresponding length of FLEX Drain PVC (Amerimax Home Products, Inc., Dallas, TX, USA) of diameter = 10.16 cm is cut based on the circumference of the tree. A 5.3 cm wide strip is removed from the detached PVC piping, and is curled into a ring shape (Figure 7A). When installing Aquabex, the underlying brush immediately surrounding the base of the tree is removed to create a flat surface. Soil is added between the tree trunk and the inner ring of Aquabex to eliminate gaps that could allow SLF to circumvent the trap and crawl through. Aquabex is installed, with the gap sealed using GE advanced water-resistant silicone caulk (Henkel Corporation, Rocky Hill, CT, USA). Once Aquabex is sealed, 5% ECOS Hypoallergenic Liquid Dish Soap solution is added.

### 2.6. Aquabex Field Tests and Deployment

Aquabex was tested under controlled conditions on five tree species throughout the SLF life cycle. For each test, SLF (*n* = 20) were captured, marked with UV-fluorescent dye, and released near the trunk of the tested tree. A sticky band was installed 1 m above the tree base and covered with a protective screen to minimize harm to non-target species. For each tree, the experiment was conducted twice under an identical setup and environment: first without Aquabex (Figure 7B), and then with Aquabex installed (Figure 7C).

**Figure 8:**
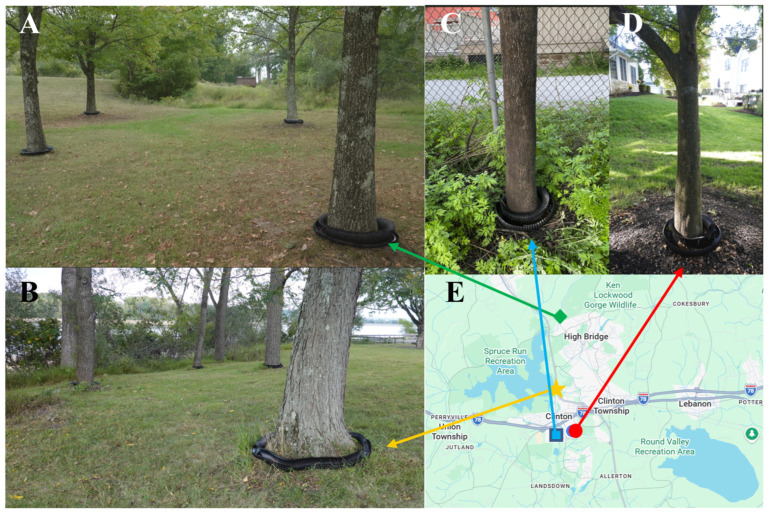
Aquabex deployment: (**A**) Site 1, Aquabex deployed at a private property in High Bridge, NJ, USA; (**B**) Site 2, Aquabex deployed near Spruce Run Reserve in Annandale, NJ, USA; (**C**) Site 3, Aquabex deployed on a TOH near Landsdown Trail; and (**D**) Site 4, Aquabex deployed at a private property in Clinton, NJ, USA. (**E**) Geographical map showing the test site locations with various icons.

Twelve Aquabex units were deployed across four sites (40.633876°, −74.912765°; 40.655188°, −74.926985°; 40.630786°, −74.902406°; 40.698037°, −74.887917°) in Hunterdon County, New Jersey, USA from 19 May to 31 May 2025: two with light SLF presence, one with medium SLF presence, and one with heavy SLF presence from previous year (Figure 8A–E). Sticky bands were installed on each tree during this period to record the number of SLF that escaped Aquabex. These trees were inspected daily throughout the deployment period.

## 3. Results and Discussion

### 3.1. Behavioral Experiments

**Figure 9:**
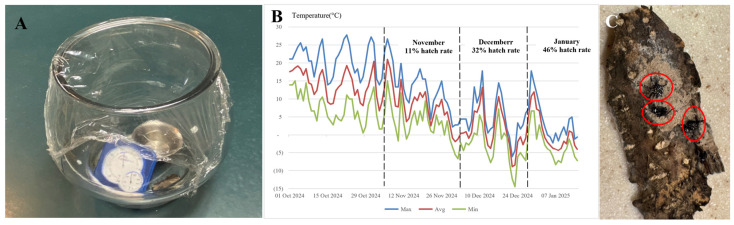
(**A**) SLF hatching environment under constant temperature of 25 °C and humidity of 70% (**B**) Daily temperature data sourced from National Centers for Environmental Information for Clinton, New Jersey, where the SLF eggs were collected. (**C**) Red circles indicate newly hatched SLF nymphs.

Freezing Period: SLF egg cohorts collected in December (32%) and January (46%) (Figure 9A,C) showed significantly higher hatch rates than the November cohort (11%) (Table 1). This is due to the requisite freezing period—the November cohort of eggs did not experience a significant freezing period, whereas the December and January cohorts experienced varied lengths (Figure 9B).

The data indicate that longer exposure to freezing temperatures extended hatching time while also increasing the overall hatching success rate. These findings suggest that SLF egg masses require a sufficient “freezing period” to achieve higher hatch rates.

**Figure 10:**
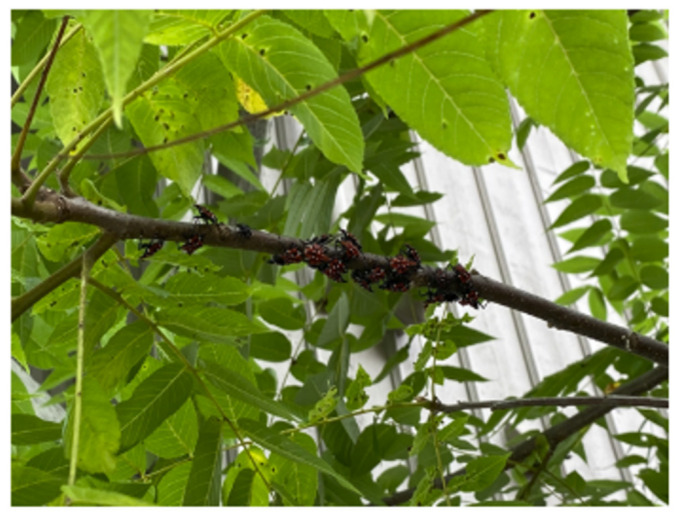
SLF nymphs clustered on a Black Walnut tree in the Clinton, NJ test site.

Clustering and Crawling: After SLF were marked and released, the bugs clustered on two of the seven available branches, exhibiting non-random clustering (*p* << 0.001) (Table 2). All six experiments conducted across three species of trees indicated statistically significant clustering behavior (Table 3). Among repeated experiments on the same tree, SLF individuals consistently exhibited aggregation behaviors, even when choosing different branches. This aggregation pattern suggests that clustering is driven by behavioral mechanisms rather than branch-specific characteristics. SLF display distinct clustering behavior (Figure 10) that plays an important role in survival, reproduction, and ecological impact. This clustering behavior is visible across all life stages and is driven by a mix of chemical signaling, host preference, environmental factors, and behavioral traits.

Four hours after release, 88% (Table 4) of the SLF had returned to their habitat tree, and all individuals did so by crawling up the trunk, showing that crawling, instead of jumping, is the primary mode of travel.

Jumping: SLF only jump when they perceive danger or encounter obstacles which they are unable to crawl through. First and second-stage nymphs were unable to overcome the barrier set at angles of 45° or greater (Table 5). Larger instar nymphs were able to escape at wider barrier angles (Table 5). Adult SLF exhibited significantly increased escape capabilities due to their ability to glide using their wings (Table 5).

**Figure 11:**
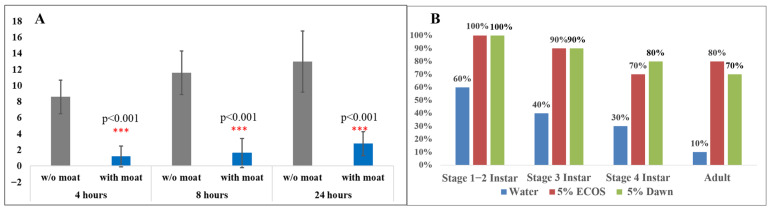
(**A**) Experimental results: the water moat significantly reduces the chance that SLF reach TOH branch. *** indicates *p* < 0.001. (**B**) Percentage of SLF that drown within one minute, by life stage.

Hydrophobicity: In the absence of the water moat, SLF were strongly attracted to the TOH; however, the presence of the water moat effectively deterred SLF invasion (Figure 11A). Many individuals remained at the bottom or sides of the enclosure without attempting to cross the moat. Some attempted to leap from the edge of the plate toward the branch, but most were unable to grip the sides and ultimately drowned. From these experimental results, it can be concluded that SLF exhibit strong aversion to water.

SLF, especially 1st–2nd instar nymphs, have very low survival rates in water (Figure 11B). In regular water, 60% of all nymphs drowned within one minute, and using 5% dish detergent solutions increased mortality to 100%. Later-stage instars and adult SLF, on average, survived longer in both water and 5% dish detergent solutions, due to their greater strength and ability to resist fatigue. The detergent lowered surface tension, impairing the SLF’s ability to escape and leading to more rapid mortality.

The hatching experiments demonstrate that SLF egg masses require a freezing period for successful hatching, which can be applied to improving current prediction models. Their clustering behavior makes them more readily identifiable by an object-detection model. SLF’s crawling, jumping abilities, and aversion to water can be applied in developing a new trap.

### 3.2. Prediction Model Results

This research used SLF distributions in the U.S. to develop a MAXENT prediction model, predicting high suitability for SLF proliferation in China, Korea, Japan, and the United States. Within the U.S., the model forecasts SLF establishment on the East Coast, Midwest, and portions of the South. In comparison, Wakie et al. projects a global distribution across six continents, with a high likelihood of SLF invasion in the Mediterranean, Europe, and the U.S. West Coast.

**Figure 12:**
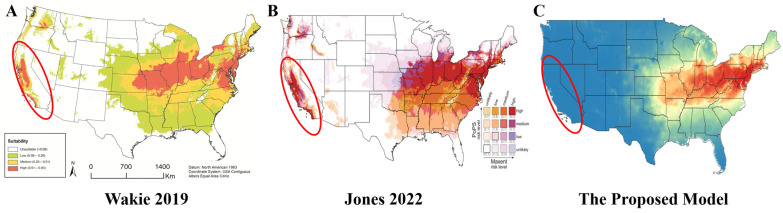
Unlike previous models from (**A**) [36] and (**B**) [7], (**C**) AI-LyD suggests that the West Coast, particularly California (circled), is unlikely to face SLF invasion, consistent with current SLF proliferation trends. Panels (**A**,**B**) are reproduced from [36] and [7], respectively, under the Creative Commons Attribution (CC BY) license.

Although the AUC of this research (0.821) is slightly lower than the 0.89 reported by Wakie et al., this research correctly predicts a low chance of SLF infestation in the Mediterranean and US West Coast regions, explaining the “California Paradox.” (Figure 12). This presented model demonstrates a higher sensitivity (0.888 vs. 0.80), indicating a greater ability to correctly identify areas at risk of invasion. The Kappa statistic (0.642) is comparable to Wakie et al.’s 0.67. This improved model provides macro-level guidance to where prevention resources should be prioritized.

As Table 6 shows, the model’s performance is primarily driven by the presence of a freezing period contributing the most to the model’s training, emphasizing the critical role of a necessary cold period for successful SLF proliferation identified in 3.1. Precipitation and temperature seasonality are more significant than the presence of TOH, indicating that climate conditions play a larger role in determining SLF proliferation viability.

### 3.3. Detection Model Results

The model was tasked with identifying SLF from two sets of 50 low-fidelity images reflecting real-world background and lighting conditions. One set contained clustered SLF, while the other only contained 1–2 SLF.

**Figure 13:**
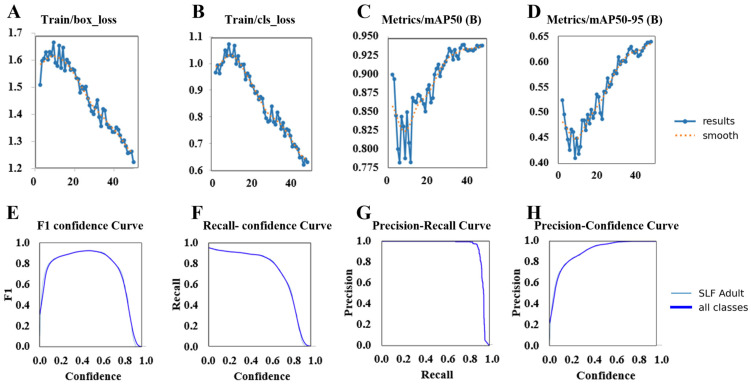
YOLO11 model’s training and performance metrics. (**A**,**B**) Training loss curves show improving box and class losses (**C**,**D**) Improvements reflected in mAP50 and mAP50-95 accuracy metrics (**E**–**H**) Performance evaluation curves across different SLF life stages.

**Figure 14:**
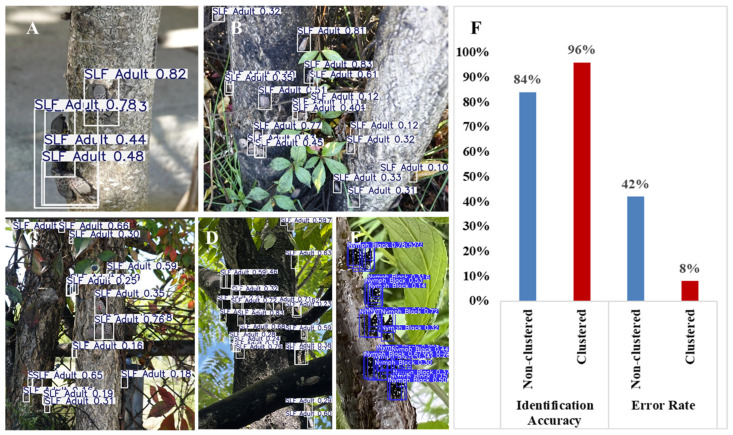
(**A**–**F**) SLF detection results from the clustered dataset demonstrate high confidence in its predictions. (**B**) Accuracy rate and error rate of detection models. Clustered SLF are more likely to be identified and with lower error rates.

The performance metrics (Figure 13A–H) suggest that the SLF’s clustering behavior serves to enhance the performance of the detection module. As the dataset of individual spotted lanternflies were derived from the clustered images, differences in detection performance are unlikely to be a result of variations in lighting, scale, or background. Instead, SLF aggregation increases the visual density of their spots (Figure 14A–E), increasing detection accuracy even under suboptimal conditions. The natural redundancy of SLF patterns reduces false positive detections (Figure 14F), strengthening the detection model’s reliability in real-world scenarios. The enhanced model therefore supports drone-mediated monitoring, as aggregation patterns are more reliably detected from greater distances or with lower-resolution imaging.

### 3.4. Aquabex Design, Deployment and AI Implementation

**Figure 15:**
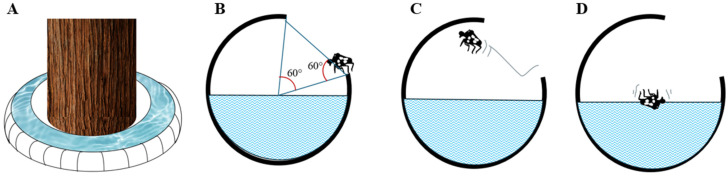
Aquabex’s design. (**A**) 3-D view of Aquabex. (**B**–**D**): Cross-sectional sequence of SLF jumping into Aquabex.

Figure 15A–D shows the design of the Aquabex device. The 60° opening angle was designed to prevent SLF individuals from escaping by jumping, based on experimental results indicating that the jumping angles of Stage 1–4 instars are generally below 60° (see Table 5, SLF jumping experiment). The Aquabex design effectively prevents most instars from reaching the protected tree trunk.

**Figure 16:**
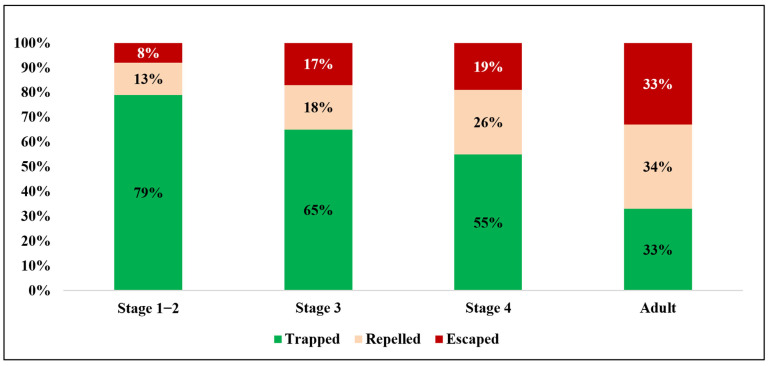
Aquabex’s efficiency in field comparison test.

Twenty experiments were conducted on five tree species—Tree of Heaven (TOH), Japanese snowbell, black walnut, maple, and boxelder (*Acer negundo*)—across all four SLF instar stages and the adult stage. Experimental results in Figure 16 show that Aquabex is highly effective in trapping and repelling Stage 1–2 SLF nymphs, with an efficiency rate of 92% (95% CI: 85.0–95.9%). Aquabex maintains strong performance against later nymphal stages, with 83% effectiveness (95% CI: 74.5–89.1%) and 81% effectiveness (95% CI: 72.2–87.5%) for Stage 3 and 4 nymphs, respectively. The overall efficiency for SLF nymph is 85% (95% CI: 80.9–88.9%). For adult SLF, the efficiency rate remains substantial at 67% (95% CI: 57.3–75.4%) (Figure 16).

**Figure 17:**
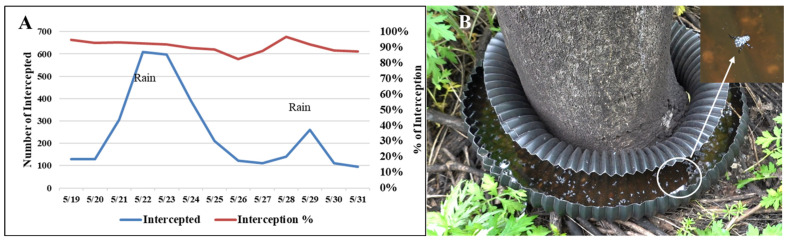
(**A**) Field test results from 19 May 2025 to 31 May 2025 of 12 Aquabex placed across 4 sites in Clinton, NJ (**B**) Aquabex capturing SLF.

During the 13-day field trial period, which coincided with the SLF hatching season and peak first-instar activity, Aquabex demonstrated strong effectiveness. Across 12 Aquabex on four sites, 3212 or 91% (95% CI: 90.1–92.0%) of SLF (Figure 17A) were captured and drowned in the Aquabex barrier (Figure 17B). In contrast, comparable trees of the same species without Aquabex experienced similar levels of SLF invasion, indicating that the reduction in SLF numbers on treated trees was due to the barrier rather than lower SLF volume.

This research also found a correlation between the number of SLF captured and periods of inclement weather. Capture numbers were higher following rainfall, suggesting that SLF nymphs were displaced by the rain. Periodic rainfall serves to naturally refill Aquabex, thereby requiring less regular maintenance. In some locations, such as Aquabex-5, Aquabex-7 and Aquabex-10, following the 13-day deployment, SLF populations were extremely sparse.

It is noted that Aquabex is more effective against the smaller instar stages due to their smaller size relative to the contraption itself. Larger SLF instar stages, on the other hand, may possess sufficient jumping power to overcome the moat, thereby reducing the number of SLF eliminated in the later stages. Modifications may be conducted to these portions to increase coverage of the later life stages of SLF.

The use of a 5% eco-friendly dish detergent solution in Aquabex warrants the consideration of environmental impact. While the presence of detergent is noted to increase SLF mortality in controlled experiments (see Figure 11), its potential for runoff and non-target effects may need to be evaluated for broader deployment. However, in our field trials, there were no other insect species that were affected by Aquabex deployment. No animals were also observed to have interacted with the Aquabex trap during the field deployments. Aquabex is designed to accommodate for storing rainwater and limiting any runoff. However, heavy rainfall could potentially cause minor runoff, and future deployments will consider placement away from sensitive habitats. Compared to conventional pesticides, the detergent solution is a far lower ecological burden. Future work will investigate other biodegradable solutions for Aquabex use.

Most importantly, the materials required for Aquabex fabrication are commercially available in the market. The estimated production cost for Aquabex is approximately USD $0.48 per unit, with the potential for further cost reduction through large-scale manufacturing. This low manufacturing cost supports broad adoption and widespread deployment of Aquabex in agricultural and ecological settings.

### 3.5. AI-Supported Targeted Prevention and Elimination Strategies

**Figure 18:**
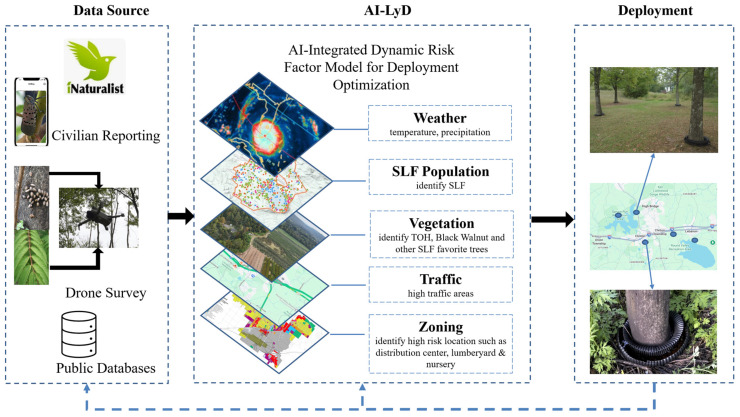
AI-LyD’s AI-driven Aquabex deployment plan, integrating multilayered spatial data for optimized mitigation.

AI-LyD’s feedback structure operates through refined update cycles and decision rules that connect prediction, detection and deployment (Figure 18). The initial prediction model is trained on existing environmental and ecological datasets (e.g., host tree density, freezing-period presence) to generate a baseline risk map. Data from drone surveys and citizen-science field observations is then processed through the detection model to determine SLF presence. AI-LyD then generates a spatially optimized deployment map of Aquabex, prioritized by SLF density from the detection model and threat to agriculture and natural resources. New infestation locations and sites where SLF populations have been eliminated are incorporated into the prediction dataset on a weekly basis, and the model is retrained to reflect the new distribution trend. Additionally, uploaded images are then used in further training the detection model. Data latency is primarily determined by the frequency of drone surveys and citizen-science reporting; however, this bottleneck can be overcome by increasing SLF awareness to vulnerable regions identified in the prediction model and deploying more low-cost drones. The AI-LyD approach creates a scalable, interconnected system: prediction guides monitoring focus, detection validates and updates spatial risk predictions, and deployment is adjusted based on recent observational inputs, allowing for resource-efficient SLF mitigation.

### 3.6. Limitations

A limitation of the freezing-period behavioral experiment is that the egg cohorts were collected by month as a proxy for varying durations of cold exposure, rather than subjecting egg cohorts to controlled temperature and time treatments. Although the findings provide directional evidence supporting the importance of a freezing period in SLF hatching success, they do not indicate a critical temperature threshold or exact exposure duration. Future investigations will control the freezing period length and temperatures to optimize overwintering temperature and duration.

As this research was conducted primarily in Central New Jersey, the current training of the AI-LyD model may not capture the full ecological and climatic variability present across the broader U.S. range of SLF invasion. Differences in background texture, lighting, and host tree characteristics could reduce detection accuracy. To improve model generalizability, future work should expand the training dataset to include images from varied habitats—encompassing multiple tree species, ground surfaces, and artificial structures—and apply transfer learning and cross-domain augmentation techniques to enhance robustness across heterogeneous landscapes.

While Aquabex proves to be an effective device in SLF population control and invasion prevention, its design can still undergo iterative optimization (ex. geometry, materials). Test sites are limited in the initial proof-of-concept stage. Larger deployments across diverse geographic regions and bioclimatic conditions are needed to further validate effectiveness and scalability. Future works will focus on expanding the number of deployment sites across various regional vegetations and climates.

## 4. Conclusions

This research presents AI-LyD, an AI-driven systemic solution to SLF invasion, integrating behavioral analysis into proliferation modeling, object detection, and AI-integration elimination methods. A set of five behavioral experiments evaluated the minimum required freezing period for SLF eggs for successful hatching, clustering patterns, crawling tendencies, jumping trajectories, and hydrophobicity. When the egg freezing period below 4.44 °C sustained over three months and SLF hydrophobicity factors were incorporated into the MAXENT model, these parameters improved prediction (AUC = 0.821, Sensitivity = 0.888, Kappa Statistic = 0.642) and resolved inconsistencies in prior models such as the “California Paradox.” The updated model predicts future invasion in the Midwestern and Southeastern states. Temperature and weather extremes accompanying climate change may render formerly SLF-inhospitable geographical areas amenable to sufficient overwintering and hatching. The YOLOv11-based detection software achieved a true-positive rate of 96% with a false-positive rate of 8% in real-world lighting and substrate conditions. Cluster-based training improved detection, supporting drone-based and citizen-science detection applications. Leveraging insights from SLF hydrophobicity and jumping-angle (60° ± 5°) studies, this research introduces Aquabex, a low-cost, passive water-moat trap. Laboratory and field trials (*n* = 4 sites, Hunterdon County, NJ) demonstrated 85% (95% CI: 80.9–88.9%) nymph deterrence and 67% (95% CI: 57.3–75.4%) adult reduction, culminating in a 91% (95% CI: 90.1–92.0%) population reduction after multi-week deployment. Together, the AI-LyD framework presents three major contributions to SLF management: (1) the integration of experimentally derived behaviors into predictive modeling to improve forecast accuracy; (2) the development of a field-ready AI detection system; and (3) the design and validation of a low-cost, behavior-informed trap that is powered by a dynamic AI-powered deployment system. By linking these components in a feedback loop, AI-LyD establishes a dynamic and adaptive paradigm for combating and preventing SLF populations, reduces labor and environmental costs, and opens a new frontier of integrating behavioral findings and AI-powered softwares in IPM, agriculture, and conservation.

## Figures and Tables

**Table 1:** Result of SLF egg hatching experiment.

Hatching Start Date	No. Eggs Collected	No. Eggs Hatched	Hatch Rate	Average Hatching Duration (Days)
6 November 2024	153	17	11%	25
6 December 2024	135	43	32%	31
6 January 2025	141	65	46%	35

**Table 2:** Representative sample of the SLF clustering experiment.

Experiment No.	Number of Observed SLF (O)	Number of Expected SLF (E)	(O-E)^2^/E
Branch 1	18	4.29	43.89
Branch 2	5	4.29	0.12
Branch 3	2	4.29	1.22
Branch 4	0	4.29	4.29
Branch 5	1	4.29	2.52
Branch 6	1	4.29	2.52
Branch 7	0	4.29	4.29
Total	27	30	
$\chi^{2}$			53.83
Degrees of Freedom (df)			6
*p*-Value			<0.0001

**Table 3:** Summary of Chi-square significance test analyzing SLF clustering behavior across different host tree species.

Experiment No.	Tree Species	No. Observed SLF	No. Expected SLF	$\chi^{2}$	df	*p*-Value	Significance
1	*Styrax japonicus*	27	30	58.83	6	<0.01	***
2		26	30	20.00	6	<0.01	**
3	*Juglans nigra*	25	30	87.10	16	<0.001	***
4		28	30	150.80	16	<0.001	***
5	*Acer rubrum*	27	30	28.33	9	<0.001	***
6		25	30	22.33	9	<0.01	**

** indicates *p* < 0.01; *** indicates *p* < 0.001.

**Table 4:** Summary of SLF return rates across experiments. The tree trunk serves as the primary pathway for SLF to return to their habitat.

	Experiment 1	Experiment 2	Experiment 3	Experiment 4	Experiment 5	Experiment 6	Total
Released	30	30	30	30	30	30	180
Returned	27	26	25	28	27	25	158
Return Rate	90%	87%	83%	93%	90%	83%	88%

**Table 5:** Percentage of SLF that overcame the artificial barrier at varying angles, by life stage.

	Stage 1–2 Instar	Stage 3 Instar	Stage 4 Instar	Adult
<30°	77%	70%	53%	40%
<45°	97%	83%	73%	57%
<60°	100%	93%	87%	70%

**Table 6:** Percent contribution of various environmental layers.

Factor	Percent Contribution	Permutation Importance
Presence of Freezing Period	59.3%	17.6%
Precipitation During Warmest Quarter	26.5%	18.9%
Temperature Seasonality	5.4%	46.0%
Presence of TOH	2.7%	7.5%
Total annual precipitation	0.7%	9.9%

## Data Availability

Bioclimatic variables were sourced from the WorldClim online database. SLF and TOH presence point data was sourced from iNaturalist and the Global Biodiversity Information Facility (GBIF Occurrence Download. Available online: https://www.gbif.org/species/5157899 and https://www.gbif.org/species/3190653, accessed on 20 February 2026). Geographic data was sourced from US Geological Services. Further inquiries regarding the raw data supporting the contributions presented in this research can be directed to the corresponding author.

## Abbreviations

The following abbreviations are used in this manuscript:

SLF	Spotted Lanternfly (*Lycorma delicatula*)
TOH	Tree of Heaven (*Ailanthus altissima*)
IPM	Integrated Pest Management System
AI	Artificial Intelligence
ML	Machine Learning
YOLO	You Only Look Once (Computer Vision Model)

## References

[1] Ladin Z.S., Eggen D.A., Trammell T.L.E., D’Amico V. (2023). Human-Mediated Dispersal Drives the Spread of the Spotted Lanternfly (*Lycorma delicatula*). Sci. Rep..

[2] Meurisse N., Rassati D., Hurley B.P., Brockerhoff E.G., Haack R.A. (2019). Common Pathways by Which Non-Native Forest Insects Move Internationally and Domestically. J. Pest Sci..

[3] Barringer L.E., Donovall L.R., Spichiger S.-E., Lynch D., Henry D. (2015). The First New World Record of *Lycorma delicatula* (Insecta: Hemiptera: Fulgoridae). Entomol. News.

[4] Cornell University Spotted Lanternfly Reported Distribution Map. https://cals.cornell.edu/integrated-pest-management/outreach-education/whats-bugging-you/spotted-lanternfly/spotted-lanternfly-reported-distribution-map.

[5] Kim H., Kim S., Lee Y., Lee H.-S., Lee S.-J., Lee J.-H. (2021). Tracing the Origin of Korean Invasive Populations of the Spotted Lanternfly, *Lycorma delicatula* (Hemiptera: Fulgoridae). Insects.

[6] Urban J.M. (2020). Perspective: Shedding Light on Spotted Lanternfly Impacts in the USA. Pest Manag. Sci..

[7] Jones C., Skrip M.M., Seliger B.J., Jones S., Wakie T., Takeuchi Y., Petras V., Petrasova A., Meentemeyer R.K. (2022). Spotted Lanternfly Predicted to Establish in California by 2033 without Preventative Management. Commun. Biol..

[8] Harner A.D., Leach H.L., Briggs L., Centinari M. (2022). Prolonged Phloem Feeding by the Spotted Lanternfly, an Invasive Planthopper, Alters Resource Allocation and Inhibits Gas Exchange in Grapevines. Plant Direct.

[9] Lavely E., Iavorivska L., Uyi O., Eissenstat D.M., Walsh B., Primka E.J., Harper J., Hoover K. (2022). Impacts of Short-Term Feeding by Spotted Lanternfly (*Lycorma delicatula*) on Ecophysiology of Young Hardwood Trees in a Common Garden. Front. Insect Sci..

[10] Barringer L., Ciafré C.M. (2020). Worldwide Feeding Host Plants of Spotted Lanternfly, With Significant Additions From North America. Environ. Entomol..

[11] Harper J.K., Stone W., Kelsey T.W., Kime L.F. (2019). Potential Economic Impact of the Spotted Lanternfly on Agriculture and Forestry in Pennsylvania.

[12] Liu H. (2022). Oviposition Selection in Spotted Lanternfly: Impact of Habitat and Substrate on Egg Mass Size and Hatchability. Front. Insect Sci..

[13] Liu H. (2019). Oviposition Substrate Selection, Egg Mass Characteristics, Host Preference, and Life History of the Spotted Lanternfly (Hemiptera: Fulgoridae) in North America. Environ. Entomol..

[14] Penn State Extension Spotted Lanternfly Management Guide. https://extension.psu.edu/spotted-lanternfly-management-guide.

[15] Urban J.M., Leach H. (2023). Biology and Management of the Spotted Lanternfly, *Lycorma delicatula* (Hemiptera: Fulgoridae), in the United States. Annu. Rev. Entomol..

[16] Li C., Xu A.J., Beery E., Hsieh S.T., Kane S.A. (2023). Putting a New Spin on Insect Jumping Performance Using 3D Modeling and Computer Simulations of Spotted Lanternfly Nymphs. J. Exp. Biol..

[17] Dara S.K., Barringer L., Arthurs S.P. (2015). *Lycorma delicatula* (Hemiptera: Fulgoridae): A New Invasive Pest in the United States. J. Integr. Pest Manag..

[18] Leach H., Walsh B., Urban J. (2021). Evaluation of Insecticides for Control of Spotted Lanternfly in Ornamental Nursery Crop, 2019. Arthropod Manag. Tests.

[19] Bexfield L.M., Belitz K., Lindsey B.D., Toccalino P.L., Nowell L.H. (2021). Pesticides and Pesticide Degradates in Groundwater Used for Public Supply across the United States: Occurrence and Human-Health Context. Environ. Sci. Technol..

[20] Gunstone T., Cornelisse T., Klein K., Dubey A., Donley N. (2021). Pesticides and Soil Invertebrates: A Hazard Assessment. Front. Environ. Sci..

[21] Elmquist J., Biddinger D., Phan N.T., Moural T.W., Zhu F., Hoover K. (2023). Potential Risk to Pollinators from Neonicotinoid Applications to Host Trees for Management of Spotted Lanternfly, *Lycorma delicatula* (Hemiptera: Fulgoridae). J. Econ. Entomol..

[22] Leska A., Nowak A., Nowak I., Górczyńska A. (2021). Effects of Insecticides and Microbiological Contaminants on Apis Mellifera Health. Molecules.

[23] Clifton E.H., Hajek A.E., Jenkins N.E., Roush R.T., Rost J.P., Biddinger D.J. (2020). Applications of *Beauveria bassiana* (Hypocreales: Cordycipitaceae) to Control Populations of Spotted Lanternfly (Hemiptera: Fulgoridae), in Semi-Natural Landscapes and on Grapevines. Environ. Entomol..

[24] Hajek A.E., Everest T.A., Clifton E.H. (2023). Accumulation of Fungal Pathogens Infecting the Invasive Spotted Lanternfly, *Lycorma delicatula*. Insects.

[25] Gómez Marco F., Hoddle M.S. (2024). Proactive Biological Control of Spotted Lanternfly: Parasitism and Host Feeding Behavior of *Anastatus orientalis* (Hymenoptera: Eupelmidae) on *Lycorma delicatula* (Hemiptera: Fulgoridae) Egg Masses. Biol. Control.

[26] iNaturalist Spotted Lanternfly (*Lycorma delicatula*). https://www.inaturalist.org/taxa/324726-Lycorma-delicatula.

[27] Cornell University Spotted Lanternfly Management. https://cals.cornell.edu/integrated-pest-management/outreach-education/whats-bugging-you/spotted-lanternfly/spotted-lanternfly-management.

[28] New Jersey Department of Agriculture Spotted Lanternfly. https://www.nj.gov/agriculture/divisions/pi/prog/pests-diseases/spotted-lanternfly/.

[29] Cornell University Spotted Lanternfly Damage. https://cals.cornell.edu/integrated-pest-management/outreach-education/whats-bugging-you/spotted-lanternfly/spotted-lanternfly-damage.

[30] Hearon L.E. (2020). What’s the Catch? Collateral Mortality of Spotted Lanternfly Sticky Banding. https://repository.upenn.edu/server/api/core/bitstreams/3b2d6013-998a-4ffd-86e0-0456c8711d25/content.

[31] Francese J.A., Cooperband M.F., Murman K.M., Cannon S.L., Booth E.G., Devine S.M., Wallace M.S. (2020). Developing Traps for the Spotted Lanternfly, *Lycorma delicatula* (Hemiptera: Fulgoridae). Environ. Entomol..

[32] Cooperband M.F., Murman K.M. (2025). Improving Traps for Spotted Lanternflies, *Lycorma delicatula* (Hemiptera: Fulgoridae), by Leveraging Their Own Signals. Insects.

[33] Strömbom D., Pandey S. (2021). Modeling the Life Cycle of the Spotted Lanternfly (*Lycorma delicatula*) with Management Implications. Math. Biosci..

[34] Barker B.S., Beyer J., Coop L. (2025). Real-Time Integrative Mapping of the Phenology and Climatic Suitability for the Spotted Lanternfly, *Lycorma delicatula*. Insects.

[35] Lewkiewicz S.M., Seibold B., Helmus M.R. (2024). Quantifying Population Resistance to Climatic Variability: The Invasive Spotted Lanternfly Grape Pest Is Buffered against Temperature Extremes in California. Ecol. Model..

[36] Wakie T.T., Neven L.G., Yee W.L., Lu Z. (2019). The Establishment Risk of *Lycorma delicatula* (Hemiptera: Fulgoridae) in the United States and Globally. J. Econ. Entomol..

[37] Keena M.A., Hamilton G., Kreitman D. (2023). The Potential Climatic Range of Spotted Lanternfly May Be Broader than Previously Predicted. Front. Insect Sci..

[38] Bai Q., Gao R., Li Q., Wang R., Zhang H. (2024). Recognition of the Behaviors of Dairy Cows by an Improved YOLO. Intell. Robot..

[39] Belouard N., Behm J.E. (2023). Spotted! Computer-Aided Individual Photo-Identification Allows for Mark-Recapture of Invasive Spotted Lanternfly (*Lycorma delicatula*). Front. Insect Sci..

[40] Krizhevsky A., Sutskever I., Hinton G.E., Pereira F., Burges C.J., Bottou L., Weinberger K.Q. (2012). ImageNet Classification with Deep Convolutional Neural Networks. Proceedings of the Advances in Neural Information Processing Systems.

[41] Fuller A.K., Augustine B.C., Clifton E.H., Hajek A.E., Blumenthal A., Beese J., Hurt A., Brown-Lima C.J. (2024). Effectiveness of Canine-assisted Surveillance and Human Searches for Early Detection of Invasive Spotted Lanternfly. Ecosphere.

[42] Papakie K. (2023). CMU Team Develops Autonomous Robot to Stave Off Spotted Lanternflies. https://www.cmu.edu/news/stories/archives/2023/june/cmu-team-develops-autonomous-robot-to-stave-off-spotted-lanternflies.

[43] Zhang S. (2024). ArTreeficial: An AI-Tree Controlling Spotted Lanternfly Populations Using Computer Vision and Dynamic Response. Proceedings of the 2023 IEEE International Conference on Advances in Data-Driven Analytics and Intelligent Systems (ADACIS), Marrakesh, Morocco, 23–25 November 2023.

[44] Phillips S.J., Anderson R.P., Schapire R.E. (2006). Maximum Entropy Modeling of Species Geographic Distributions. Ecol. Model..

[45] Global Biodiversity Information Facility (GBIF) (2025). *Lycorma delicatula* (White, 1845). https://doi.org/10.15468/dl.jjrmzr.

[46] Global Biodiversity Information Facility (GBIF) (2025). *Ailanthus* *altissima*. https://doi.org/10.15468/dl.byq4h5.

[47] Jocher G. Ultralytics YOLO11. https://docs.ultralytics.com/models/yolo11/.

